# Maternal Haplotypes in *DHFR* Promoter and *MTHFR* Gene in Tuning Childhood Acute Lymphoblastic Leukemia Onset-Latency: Genetic/Epigenetic Mother/Child Dyad Study (GEMCDS)

**DOI:** 10.3390/genes10090634

**Published:** 2019-08-22

**Authors:** Veronica Tisato, Paola Muggeo, Tracy Lupiano, Giovanna Longo, Maria Luisa Serino, Massimo Grassi, Ermanno Arcamone, Paola Secchiero, Giorgio Zauli, Nicola Santoro, Donato Gemmati

**Affiliations:** 1 Department of Morphology, Surgery & Experimental Medicine and LTTA Centre, University of Ferrara, 44121 Ferrara, Italy; 2 Department of Pediatric Oncology & Hematology, University Hospital Policlinico, 70124 Bari, Italy; 3 Department of Biomedical & Specialty Surgical Sciences, and Centre Haemostasis & Thrombosis, University of Ferrara, 44121 Ferrara, Italy; 4 Center of Gender Medicine, University of Ferrara, 44121 Ferrara, Italy

**Keywords:** Childhood Acute Lymphoblastic Leukemia, ALL onset, *DHFR*, *MTHFR*, polymorphism/gene variant, epigenetics, parent-origin effect (POE), mother-fetus in utero crosstalk, folate, MTX

## Abstract

Childhood acute lymphoblastic leukemia (ALL) peaks around age 2–4, and in utero genetic epigenetic mother-fetus crosstalk might tune ALL onset during childhood life. Folate genes variably interact with vitamin status on ALL risk and prognosis. We investigated *DHFR* and *MTHFR* gene variants in 235 ALL children and their mothers to disclose their role in determining ALL onset age and survival. Pyrosequence of *DHFR* 19bp ins/del (rs70991108; W/D), *MTHFR* C677T (rs1801133; C>T), and *MTHFR* A1298C (rs1801131; A>C) was assessed in children and in 72% of mothers for dyad-analysis comparison. *DHFR* DD-children had delayed ALL onset compared to WW-children (7.5 ± 4.8 vs. 5.2 ± 3.7 years; *P* = 0.002) as well as *MTHFR* 1298 CC-children compared to AA-children (8.03 ± 4.8 vs. 5.78 ± 4.1 years; *P* = 0.006), and according to the strong linkage disequilibrium between *MTHFR* 677 T-allele and 1298C-allele, *MTHFR* TT-children showed early mean age of onset though not significant. Offspring of *MTHFR* 677 TT-mothers had earlier ALL onset compared to offspring of 677 CC-mothers (5.4 ± 3.3 vs. 7 ± 5.3 years; *P* = 0.017). *DHFR*/*MTHFR 677* polymorphism combination influenced onset age by comparing DD/CC vs. WW/TT children (8.1 ± 5.7 vs. 4.7 ± 2.1 years; *P* = 0.017). Moreover, mother-child genotype combination gave 5.5-years delayed onset age in favor of DD-offspring of 677 CC-mothers vs. WW-offspring of 677 TT-mothers, and it was further confirmed including any D-carrier children and any 677 T-carrier mothers (*P* = 0.00052). Correction for multiple comparisons maintained statistical significance for *DHFR* ins/del and *MTHFR* A1298C polymorphisms. Unexpectedly, among the very-early onset group (<2.89 years; 25th), DD-genotype inversely clustered in children and mothers (4.8% vs. 23.8% respectively), and accordingly ALL offspring of homozygous DD-mothers had increased risk to have early-onset (adjusted OR (odds ratio) = 3.08; 1.1–8.6; *P* = 0.03). The opposite effect *DHFR* promoter variant has in tuning ALL onset-time depending on who is the carrier (i.e., mother or child) might suggest a parent-origin-effect of the D-allele or a two-faced epigenetic role driven by unbalanced folate isoform availability during the in-utero leukemogenesis responsible for the wide postnatal childhood ALL latency.

## 1. Introduction

Acute lymphoblastic leukemia (ALL) is the most common pediatric cancer, nearly, accounting 80% of childhood cancers [1]. Epidemiological data report 2900 new diagnoses of childhood ALL per year in the USA [2] and about 50 new cases per million per year in Europe [3], with different ethnicities sharing such a high incidence [4].

Pediatric ALL (i.e., patients <18 years) peaks between 2 and 4 years of age, and genetics and environment mutually interact in cancer etiopathogenesis. Besides, the maternal in utero environment necessarily plays a key role allowing during pregnancy a mutual mother/embryo genetic and epigenetic cross-talk. Moreover, maternal genetic background and lifestyle might act on the developing fetus affecting in utero hematopoiesis and the age of ALL onset during childhood life [5,6].

Some studies investigated the role of gene variants (Single Nucleotide Polymorphism; SNP) or copy number variations (CNV) in selected genes determining the time of leukemic onset with particular attention toward ALL in early age [7,8,9]. Moreover, it has been reported that the risk of infant ALL associated with spontaneous leukemia fusion gene formation may be modified by folate availability, diet, and genetics during pregnancy [10,11].

Although treatments and survival strongly improved in the recent past, pharmacogenetics and personalized medicine approaches are mandatory and should be primarily applied to tailor medical decisions, particularly in complex diseases, such as pediatric cancers, characterized by a strong gene-environment origin. Accordingly, the current five years overall survival rate exceeds 80–85% [1,12,13], but chemotherapy variably affects the establishment of early and late adverse effects [14].

Among the common genetic factors contributing to the extreme clinical variability in terms of risk to develop hematological malignancies, onset age, response to treatment and survival, the genes involved in folate, and methylation pathway have an impact [15,16,17,18,19].

Polymorphisms in *DHFR* (dihydrofolate reductase) and *MTHFR* (methylene-tetrahydrofolate reductase) genes and in other folate-metabolizing genes have been investigated both in children and mother DNA as inherited predispositions to different pediatric pathological conditions [20,21,22,23,24] and as pharmacogenetics factors involved in treatment response and survival both in adult and pediatric patients [11,15,16,17,18,25,26,27]. We previously reported a significant under-representation of *DHFR* and *MTHFR* gene variants among adult ALL and non-Hodgkin’s lymphoma in combined double carrier status, deserving increased drug-related toxicity and reduced survival to the polymorphic condition [15,16,17,18].

These genes reduce and process the main intracellular folate isoforms, involved in DNA synthesis, stability, methylation, and epigenetic processes with key roles in cancer risk establishment and treatment response [19,28]. Nevertheless, coexistence of *DHFR* 19bp ins/del (rs70991108) in the promoter/first intron of the gene, *MTHFR* 677 C>T (rs1801133) in A223V codon of exon 4, and *MTHFR* 1298 A>C (rs1801131) in E429A codon of exon 7 of the gene have never been investigated in combination in a cohort of mother-child dyads of pediatric ALL. Given that any change in the activity of these enzymes can synergize in modifying the amount and availability of the intracellular folate pool isoforms, it would be crucial to investigate the effects of the genetic and epigenetic crosstalk between mother and child DNA, potentially affecting the in utero environment and leukemogenesis and in turn ALL onset time, latency, and survival.

Methotrexate (MTX), a folate antagonist, is a crucial drug in the treatment of childhood ALL, both in the consolidation phase as high dose MTX (HDMTX) and in the maintenance phase as well as in the weekly low dose administration acting via competitive inhibition of DHFR, the reducer of dihydrofolate (DHF) into tetrahydrofolate (THF). MTHFR is indeed the reducer of 5,10-methylenetetrahydrofolate (5,10-CH_2_-THF) into 5-methyl-tetrahydrofolate (5-CH_3_-THF). Both folate isoforms are essential for methylation, neo-synthesis, and stability of DNA, as well as re-methylation of toxic homocysteine into non-toxic methionine. Overall, direct MTX-mediated inhibition on DHFR also affects MTHFR pathway, by unbalancing cells of precious active folate isoforms useful for nucleic acid neo-synthesis and methylation and in contrasting cell death and apoptosis [19,28].

Functional polymorphisms into *DHFR* and *MTHFR* genes, affecting enzyme expression or activity, might unbalance availability of the intracellular folate isoforms, influencing in turn cancer risk and onset as well as in utero hematopoiesis/leukemogenesis and childhood survival.

To specifically shed light on these issues, we here focused on the role of the coexistence of *DHFR* and *MTHFR* gene variants in mother-child dyads of pediatric ALL patients treated with AIEOP (Associazione Italiana di Ematologia e Oncologia Pediatrica) protocol. 

## 2. Material and Methods

### 2.1. Study Population

The study population included 235 pediatric patients (127 males and 108 females) affected by ALL recruited at the Department of Pediatric Oncology and Hematology University-Hospital of Bari, Italy. Among the whole cohort, 204 patients had B-phenotype (B-ALL), and 27 had T-phenotype (T-ALL), and the remaining patients had mixed-phenotype acute leukemia (MPAL, *n* = 2), biphenotypic acute leukemia (BAL, *n* = 1), and acute undifferentiated leukemia (AUL, *n* = 1) (Table 1). The patients’ mothers were invited to participate in the study, and after patients or parents signed the written informed consent, we obtained blood samples from 235 patients and 169 mothers (72%). Demographic and clinical data were obtained from medical records and a detailed interview. The present study was conducted within the frame of the *GEMCDS* (Genetic/Epigenetic Mother/Child Dyad Study) aimed at recognizing any genetic and epigenetic factors with prognostic roles and effects on any aspect of childhood ALL. 

The study protocol was approved by the local ethics committee (protocol study ALL-FOLATE n. 5013). The study was conducted following the Declaration of Helsinki.

Patients received chemotherapy for ALL according to international AIEOP-BFM ALL protocols - ALL 2000–2017. Patients were grouped into standard risk (SR), intermediate-risk (IR), and high-risk (HR) group based on genetic and molecular characteristics of blasts and response to treatment.

Patients treated with ALL 2000 and ALL R2006 protocols were allocated in the HR group if t(4;11; or MLL-AF4) or t(9;22; BCR-ABL) was detected, or poor response was established after 8-day prednisone treatment, or no complete remission on day 33, or minimal residual disease (MRD) ≥5 × 10^−4^ on day 78 of treatment (MRD-HR) was assessed. Patients treated with ALL 2009 and ALL 2017 protocols were allocated in the HR group if t(4;11; or MLL-AF4), or hypodiploidia was detected, or poor response was established after 8-day prednisone treatment, or >10% blasts in the bone marrow on day 15 or no complete remission on day 33, or minimal residual disease (MRD) ≥5 × 10^−4^ on day 78 of treatment (MRD-HR) was assessed. Prednisone poor response was defined in the case of >1000 blast cells/μl in the peripheral blood after seven days of prednisone monotherapy and one injection of intrathecal MTX.

In the absence of HR criteria, patients were assigned to the IR group if they had positive MRD on day 33 and MRD on day 78 *<* 5 × 10^−4^, or were not classifiable by MRD. When MRD was negative on day 33 and day 78 with at least two markers with a sensitivity of 10^−4^ or better, patients were defined as SR [12,29]. According to ALL 2000 and R2006 protocols, SR and IR patients received four doses of HDMTX 2 g/m^2^, except children with the central nervous system or testicular involvement or T-immunophenotype at diagnosis who received four doses of 5 g/m^2^ of HDMTX. According to ALL 2009 and 2017 protocols, SR and IR patients received four doses of HDMTX at 5 g/m^2^. HR patients in all protocols received two doses of HDMTX 5 g/m^2^ in HR blocks 1 and 2. All patients received once a week dose of MTX 20 mg/m^2^ during the maintenance phase or augmented according to peripheral blood parameters.

### 2.2. Genotype Analysis

Peripheral venous whole blood was collected into Vacutainer tubes containing EDTA and stored at −20 °C. Genomic DNA was isolated from frozen whole blood by the automated DNA extraction and purification robot using magnetic beads technology (BioRobot EZ1 system QIAGEN; Hilden, Germany). *DHFR* 19bp ins/del (rs70991108; W/D), *MTHFR* C677T (rs1801133; C>T), and *MTHFR* A1298C (rs1801131; A>C) polymorphic target regions were amplified by PCR (Sure Cycler 8800 Thermocycler, Agilent Technologies, California, CA, USA) by using customized specific primers.

*MTHFR* C677T polymorphism was detected by the Pyrosequencing technique (PyroMark Q96 ID, QIAGEN) according to details shown in Table S1. Genotype confirmation was carried out by re-genotyping a random selection of samples using *HinfI* restriction analysis. The forward primer was mutagenized to create an additional (control) *HinfI* restriction site, the enzyme recognizes the rare polymorphic 677 T-allele. All restrictions were carried out according to the supplier’s instructions (NEB-cutter V2.0, New England Biolabs; Ipswich, MA, USA). There were no discrepancies among genotype detected by different methods. Known genotypes from previously published methods were used as internal sample control [30]. 

*MTHFR* A1298C polymorphism was detected by the Pyrosequencing technique (PyroMark Q96 ID, QIAGEN) according to details shown in Table S1. Genotype confirmation was carried out by re-genotyping a random selection of sample using *Fnu4HI* restriction analysis. The forward primer was mutagenized to create a novel *Fnu4HI* restriction site in presence of the C polymorphic allele. All restrictions were carried out according to the supplier’s instructions (NEB-cutter V2.0, New England Biolabs). There were no discrepancies among genotype detected by different methods. Known genotypes from previously published methods were used as internal sample control [17]. 

*DHFR* 19bp ins/del polymorphism was detected by multiplex PCR (i.e., two different allele-specific forward primers plus one common reverse primer). The final PCR products were analyzed by 8.5% PAGE ethidium bromide-stained gel to distinguish the two different alleles (*ins*-allele: W, 113bp, and *del*-allele: D, 94bp). *DHFR* genotype confirmation was carried out by re-genotyping a random selection of samples by Pyrosequencing technique (PyroMark Q96ID, QIAGEN) [22]. There were no discrepancies among genotypes detected by different methods. Known genotypes from previously published methods were used as internal sample control [31].

The specific primer strings used to amplify the amplicons for Pyrosequence or restriction, and the PCR cycle conditions are shown in Table S1.

### 2.3. Statistical Analysis

Statistical differences among groups for categorical (counting) and continuous (mean ± SD) variables were assessed by Chi-squared test and Student’s *t*-test comparisons, respectively. Yates’ correction or Fisher’s exact test was applied when appropriate. Statistical corrections for multiple testing, Bonferroni method included, were applied when appropriate. Odds Ratio (OR) and 95% confidence interval (95% CI) were used to estimate the risk associated with different genes or combinations. Kaplan-Meier method was applied for survival analysis associated to the gene variant or combination to recognize potential prognostic indicators [32], and the event-free survival (EFS) among groups was compared by using the Log-Rank test. To estimate the risk of having a poor clinical outcome associated to different gene variants or combinations, in terms of EFS, hazard risk (HR) and 95% CI were calculated between different classes of genotypes using Cox-proportionate hazards modeling, as previously described [33,34].

Events were defined as death, ALL relapse, or secondary malignancy, whichever occurred first. For those patients in continuous complete remission, survival times were censored at the date of the last control. Patients were also analyzed concerning prednisone response.

Deviation from Hardy–Weinberg equilibrium was calculated for each gene variant in groups and subgroups and different haplotypes [35]. *P*-values ≤0.05 were considered statistically significant. Analyses were performed by using SPSS Statistical Package (Version 22; SPSS Inc., Chicago, IL, USA) and Statistica software (Version 13.3) (StatSoft, Inc., Tulsa, OK, USA) and GraphPad Prism (version 5.0) (GraphPad Software, La Jolla, CA, USA).

## 3. Results

### 3.1. Clinical and Epidemiological Features of ALL Patients

Table 1 shows the main clinical and epidemiological features of patients stratified by sex. Our cohort of patients had a mean age of ALL-onset of 6.13 years, with females showing a lower mean age than males. Almost 80% of patients developed ALL before 10 years of age, without significant differences between males and females. The peak of incidence of the whole cohort was 2.9 years with females showing a slightly earlier and wider range compared to males (♀: 2.5–3.5 years and ♂: 3–3.5 years). 

Among ALL patients, 86.8% had B-phenotype without significant difference between sex; conversely, the T-phenotype was over-represented in males (*P* = 0.03). DNA index was less than 1.16 in 78.6% of the whole cohort with no differences between males and females. BCR-ABL1/t(9;22) and MLL-AF4/t(4;11) translocations were below 1.5% in the whole group, and they clustered in males and females, respectively. Computing any assessed translocation [t(9;22); t(4;11); t(9;11); t(1;19); t(12;21)], they significantly clustered among children with early ALL onset (<4.47 years), considering the 50th percentile of the onset age distribution as cut-off (36.9% vs. 9.4%; *P* < 0.00001). Final risk group assignment identified 22.5% HR, 53.2% IR, and 24.3% SR without significant differences between sexes as well as relapse and death rates (Table 1). 

**Figure 1:**
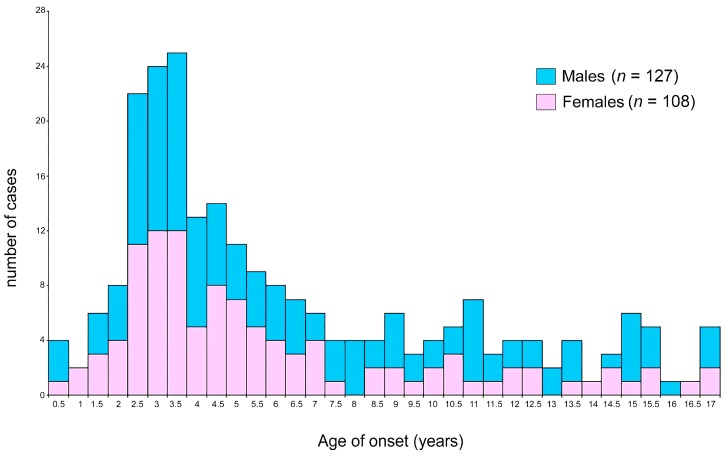
ALL onset age distribution according to sex. Females (pink) and males (light blue) in the whole cohort.

Figure 1 shows the distribution of patients age-ranked in 0.5-year increments, resulting in a comparable trend between the two sexes. Males and females overlapped in onset-age distribution, though female slightly preceded that of males (see Table 1).

### 3.2. DHFR and MTHFR Genes and ALL Age of Onset

#### 3.2.1. *DHFR* and *MTHFR* Genotype Distribution in Children and Mothers (Single Analysis)

Table 2 shows the mean onset age of ALL stratified by children genotype and by mother genotype, respectively, in the whole group of patients and the child-mother dyad subgroup.

Interestingly, *DHFR* DD-homozygous children belonging to the whole cohort (*n* = 235) showed a significant delay in ALL onset (7.5 ± 4.8 years; *P* = 0.002) when compared to WW-homozygotes (5.2 ± 3.7 years), while WD-heterozygotes showed intermediate mean age (6.7 ± 4.4 years). A subanalysis restricted to those cases belonging to mother-child dyads (*n* = 169) showed similar results (*P* = 0.007). Conversely, *DHFR* mother genotype did not show the effect on onset age.

Unexpectedly, *MTHFR* 677 variant had an opposite behavior, offspring of mother TT-homozygotes had earlier ALL onset (5.4 ± 3.3 years; *P* = 0.057) compared to offspring of mother CC-homozygotes (7 ± 5.3 years). Offspring of CT-heterozygous mothers did not differ from those of TT-homozygotes, and when they were combined, the above comparison became significant (*P* = 0.017). Finally, *MTHFR* children genotype did not show an effect on onset age. 

*MTHFR* 1298 variant showed strong significant results in the whole cohort of children, and CC-homozygotes had a significant delay in ALL onset (8.03 ± 4.8 years; *P* = 0.006) when compared with AA-homozygotes (5.7 ± 4.15 years). Any possible accidental differences in mothers’ age at delivery was excluded among *DHFR* genotype (WW: 31.6 ± 5.5; WD: 31 ± 5.7; DD: 32.5 ± 4.7 years) and *MTHFR* 1298 (AA: 31.3 ± 5.4; AC: 31.6 ± 5.6; CC: 31.4 ± 5 years), though a slightly younger mean age among *MTHFR* 677 TT-homozygous mothers was observed (29.8 ± 5; *P* = 0.021).

#### 3.2.2. *DHFR* and *MTHFR* Genotype Distribution in Children (Combined Analysis)

To define in children possible further combinations of genotypes acting on the age of onset, we stratified the whole group of ALL cases by *DHFR*/*MTHFR* genotypes (Table 3).

*DHFR*-DD genotype associated with the highest mean age of onset regardless of the *MTHFR* 677 genotype (*P* = 0.02), though the strongest effect was observed among *MTHFR* 677 CC-homozygotes and *MTHFR* 1298 AA-homozygotes. Genotype combinations gave interesting but not significant gene-dosage effects coupling *DHFR*/*MTHFR*_677_ (DD/CC 8.1 ± 5.7, DD/CT 7.8 ± 4.8 and DD/TT 6.2 ± 3.4 years) or *DHFR*/*MTHFR*_1298_ (WW/AA 4.6 ± 3.4, WW/AC 5.3 ± 3.7 and WW/CC 6.4 ± 4.4 years). Among WD-heterozygotes, *MTHFR* 677 stratification was less evident (WD/CC 6.5 ± 4.5, WD/CT 6.6 ± 4.6, and WD/TT 5.9 ± 3.9 years), virtually null among WW-homozygotes (WW/CC 5.5 ± 3.6, WW/CT 5.1 ± 4.3, and WW/TT 4.7 ± 2.1 years). Conversely, *DHFR*/*MTHFR*_1298_ stratification maintained a gene-dosage effect among WD-heterozygotes, becoming virtually null among DD-homozygotes.

The above results, though obtained from a relatively low number of cases, support the hypothesis that *DHFR* D-allele delays while *MTHFR* 677 T-allele lowers ALL onset age. This assumption was confirmed among both *MTHFR* 677 CC-homozygotes (WW/CC 5.5 ± 3.6, WD/CC 6.5 ± 4.5, and DD/CC 8.1 ± 5.7 years; *P* = 0.034) and CT-heterozygotes (WW/CT 5.1 ± 4.3, WD/CT 6.6 ± 4.6, and DD/CT 7.8 ± 4.8 years; *P* = 0.038). The lack of significant differences in mean ALL onset age among *MTHFR* TT-homozygotes (WW/TT 4.7 ± 2.1, WD/TT 5.9 ± 3.9, and DD/TT 6.2 ± 3.4 years) confirmed, in turn, the contrasting effect *MTHFR* 677 variant carries out. *DHFR/MTHFR* 1298 stratification matched that of 677. Accordingly, by comparing the extreme *DHFR*/*MTHFR*_677_ genotype combinations (i.e., DD/CC vs. WW/TT), the mean ages of onset significantly differed (DD/CC 8.1 ± 5.7 vs. WW/TT 4.7 ± 2.1 years; *P* = 0.017), showing even stronger difference by considering *DHFR*/*MTHFR*_1298_ genotype combinations (WW/AA vs. WD+DD/CC; *P* = 0.00022). The opposite effect observed among *MTHFR* polymorphisms was mainly due to the 677T/1298C alleles strong linkage disequilibrium. Trends and gaps completely matched those of the children belonging to the dyad subgroup. In details: *MTHFR* 677 CC-homozygotes (WW/CC 4.8 ± 3.1 years, WD/CC 6.5 ± 4.8 years, and DD/CC 7.3 ± 6.1 years); *MTHFR* 677 CT-heterozygotes (WW/CT 4.93 ± 4.0 years, WD/CT 6.79 ± 4.1 years, and DD/CT 7.48 ± 4.3 years); *MTHFR* 677 TT (WW/TT 4.58 ± 2.9 years, WD/TT 5.41 ± 3.2 years, and DD/TT 6.4 ± 3.5 years). By comparing the extreme *DHFR/MTHFR*_677_ genotype combinations (i.e., DD/CC vs. WW/TT), the mean ages of onset differed (DD/CC 7.3 ± 6.1 years vs. WW/TT 4.58 ± 2.9 years; *P* = 0.10), though due to the low number of cases the statistics was not significant. The same trend emerged by considering *DHFR/MTHFR*_1298_ genotype combinations (WW/AA vs. WD+DD/CC, 7.4 ± 5.9 years vs. 9.7 ± 6.7; *P* = 0.15).

#### 3.2.3. *DHFR* and *MTHFR* Genotype Distribution in Mother-child Dyads (Combined Analysis)

The above results showing that mother’s *MTHFR* 677 genotype can influence at some extent the ALL onset (Table 2), prompted us to investigate if mother-child genotype combinations might mutually interact. By performing mother-child stratification after any *DHFR* and *MTHFR* combination, we looked at identifying which genotype grouping had the greatest impact.

Considering that DD-homozygous children had the highest mean age of ALL onset in the whole cohort (7.5 ± 4.8 years, Table 2), in the dyad subgroup (7.2 ± 4.7 years, Table 2), as well as in any offspring of 677 CC-homozygous mothers (7 ± 5.3 years, Table 2), we firstly coupled these two conditions to find out potential additive effects. As expected, this subgroup showed the highest mean age of onset (10.1 ± 6.7 years, Table S2). Because this rare condition was observed only in 3% of child/mother dyad, and considering that the *DHFR* D-allele shows a clear gene-dosage-effect in children, we also included in the analysis *DHFR* D-heterozygous children. 

The comparable higher mean age of ALL onset was also observed among children *MTHFR* 1298 CC-homozygotes (8.03 ± 4.8 years and 7.05 ± 4.8 years, respectively, in the whole cohort and dyads subgroup) while mother *MTHFR* 1298 stratification did not yield any significant result (Table 2, Table S2).

Accordingly, by comparing DD+WD-children of *MTHFR* 677 CC-homozygous mothers with the remaining counterpart (i.e., WW-children of mothers carrying at least one *MTHFR* 677 T-allele), the latter confirmed the earlier mean onset age (DD+WD_child_/CC_mother_: 7.7 ± 5.9 vs. WW_child_/CT+TT_mother_: 4.3 ± 3.4 years; *P* = 0.00052), see Table S2 for nominative mean values). This observation straightens the hypothesis that *DHFR* and *MTHFR* 677 variants have contrasting effects on the age of onset when carried by the child or mother. Indeed, *DHFR* DD-homozygous children of DD-homozygous mothers did not show the expected additive effect on the age of onset. This accounts for the significant trend observed among those WD-heterozygous mothers as the number of D-alleles increased in the genotype of children, *P* = 0.005 and *P* = 0.004 (Table S2) comparing, respectively, DD-homozygotes or any D-carriers vs. WW-homozygotes (confirmed by multiple testing correction, *P* = 0.03 and 0.024, respectively). Finally, the effect of *DHFR* D-allele on mean onset age was also confirmed among offspring of mothers *MTHFR* 1298 AA-homozygotes (WW_child_/AA_mother_ 4.79 ± 3.6, WD_child_/AA_mother_ 6 ± 3.8, and DD_child_/AA_mother_ 8.4 ± 4.8 years). Indeed, by comparing WW_child_/AA_mother_ with DD_child_/AA_mother_, a significant gap of almost four years was detected (*P* = 0.0029 by *T*-test, *P* = 0.017 by multiple testing correction).

#### 3.2.4. *DHFR* and *MTHFR* Genotype Distribution in Mother (Combined Analysis)

To confirm the hypothesized opposite effect on onset age of *DHFR* and *MTHFR* variants when carried by mothers or children, and to find out further possible consequences of mother genotype combinations on ALL onset age, mothers were stratified by *DHFR*/*MTHFR* genotypes. In contrast to what observed in children genotype, the mean age of onset decreased as the number of D-alleles increased in mothers’ genotype, and this was exclusively evident among *MTHFR* 677 CC-homozygous mothers (CC/WW 8.1 ± 5.5; CC/WD 6.4 ± 5.3; CC/DD 5.5 ± 4.9 years). 

**Figure 2:**
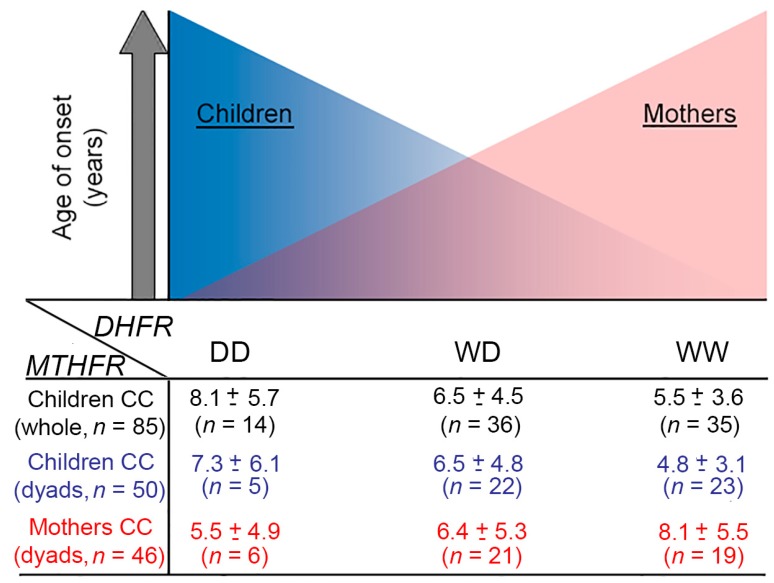
*DHFR* genotype effect on ALL (acute lymphoblastic leukemia) onset age according to the carrier. Schematic representation summarizing the hypothesized axis observed between *DHFR* gene variant and ALL onset age leading to opposite directions according to the carrier (**Upper Panel**). Mean values of ALL onset age (mean ± SD) according to *DHFR* genotype in the whole cohort (black), in the dyad-children (blue), and mothers (red) subgroup (**Lower Panel**). The *DHFR*/*MTHFR* 677 genotype combinations are referred to children or mothers genotype as indicated.

The evident opposite symmetry of the *DHFR* effect on ALL onset age according to mother or child carrier status is summarized in Figure 2 for the whole cohort and mother-child dyad. Interestingly, this was also confirmed after stratification of *DHFR* by the *MTHFR* A1298C, and being in strong linkage disequilibrium with C677T, it completely aligned with *DHFR* mean age stratification and reversed with C677T (e.g., children whole cohort: AA/WW 5.5 ± 3.3; AA/WD 6.1 ± 4.2; AA/DD 8.4 ± 5 years).

### 3.3. ALL Onset Age Distribution and DHFR/MTHFR Genotype Stratification

The ALL onset age in the whole cohort was further investigated to evaluate if the different genotypes carried by children or mothers differentially stratified by onset age quartiles (i.e., 1st ≤2.89 years; 2nd 2.9–4.47 years; 3rd 4.48–8.91 years; 4th >8.91 years). The peak-age and the first quartile of ALL onset perfectly matched, splitting our cohort into two groups: ≤2.9 years (*n* = 59, very-early ALL onset) and >2.9 years (*n* = 176), corresponding to 25% and 75% of patients cohort, respectively.

**Figure 3:**
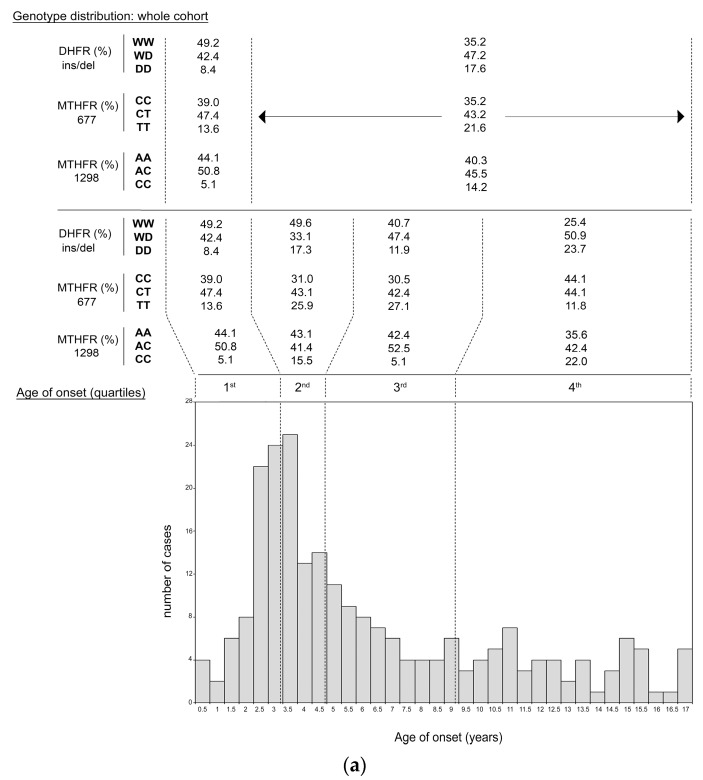

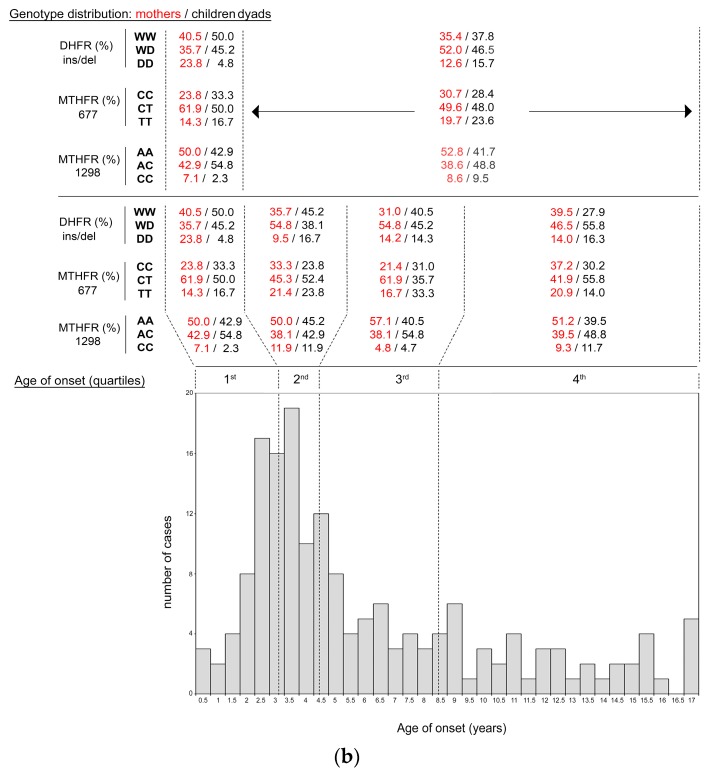
*DHFR/MTHFR* genotype distribution and ALL onset age. In panel (**a**), whole cohort of patients (*n* = 235). In panel (**b**), mother-child dyad subgroup (*n* = 196 subjects each). Genotype stratification is shown for both children and mothers in black and red, respectively. Genotype frequencies are reported as a percentage for each age quartile. Vertical dashed lines delimitate the quartiles of ALL onset age (1st, 2nd, 3rd, 4th). The number of cases (children and mothers) stratified, by *DHFR*/*MTHFR* genotypes and onset age quartiles, is shown in Table S3.

*DHFR* genotype distribution in the whole cohort of children (*n* = 235) was WW = 38.7%, WD = 46%, and DD = 15.3%, and this was according to Hardy–Weinberg equilibrium. Of note, by comparing the first quartile (very-early onset) with the fourth quartile (late-onset), the genotype distribution significantly differed (*P* = 0.01). As shown in Figure 3a, DD-homozygous children clustered in the late-onset group and were underrepresented in the very-early onset group giving significant differences (23.7% vs. 8.4%, respectively; *P* = 0.024), and this was also confirmed by comparing the very-early onset vs. the remaining cases (8.4% vs. 17.6%, respectively; *P* = 0.09). When *DHFR*-WD and -DD genotypes were combined, the extreme quartiles comparison yielded a stronger difference (*P* = 0.0077), while the comparison of the first quartile with the remaining cases gave borderline values (*P* = 0.05). Considering *MTHFR* 677 genotype distribution in the whole cohort (CC = 36.2%, CT = 44.2%, TT = 19.6%; according to Hardy–Weinberg equilibrium), *MTHFR*-TT slightly clustered in the second and third quartiles (25.9% and 27.1%, respectively), and further dedicated subanalysis did not yield significant results. Finally, within *MTHFR* 1298 genotype distribution in the whole cohort (AA = 41.3%, AC = 46.8%, CC = 11.9%; according to Hardy–Weinberg equilibrium), by comparing the first quartile with the fourth quartile, the genotype distribution significantly differed (*P* = 0.02). As shown in Figure 3a, 1298 CC-homozygous children strongly clustered in the late-onset group and were underrepresented in the very-early onset group giving significant differences (22% vs. 5.1%, respectively; *P* = 0.007), and this was also confirmed by comparing the very-early onset vs. the remaining cases (5.1% vs. 14.2%, respectively; *P* = 0.06). When *MTHFR* 1298 AC- and CC-genotypes were combined, the extreme quartiles comparison did not yield significant difference (*P* = 0.347), as well as the comparison of the first quartile with the remaining cases (*P* = 0.614) according to the recessive inheritance model of *MTHFR* 677 and 1298 polymorphisms compared to *DHFR* ins/del one (Figure 3a).

Similar results were obtained in the subset of children (*n* = 169) belonging to the mother-child dyad group (Figure 3b). As before, *DHFR*-DD genotype was underrepresented among children with very-early onset (DD = 4.8%), and when compared with the rest of patients (DD = 15.7%) or with the late-onset subgroup (DD = 16.3%), the differences were statistically significant (*P* = 0.03 and *P* = 0.04, respectively), and this was also confirmed when *DHFR*-WD and -DD genotypes were combined (*P* = 0.03). Similar to what observed in the whole cohort, *MTHFR* 677 genotype distribution analyses did not yield significant results, while *MTHFR* 1298 CC-genotype was underrepresented among children with very-early onset (CC = 2.3%), and when compared with the rest of patients (CC = 9.5%) or with the late-onset subgroup (CC = 11.7%), the differences were of borderline significant value (*P* = 0.065 and *P* = 0.057, respectively) (Figure 3b).

A complete listing, of the number and percentage of cases stratified by all the genotypes and onset age quartiles, is shown in Table S3.

In an explorative attempt, considering that the genetics of the in utero environment (mother’s genotype) might have a major role on those children who developed very-early ALL, we assessed a targeted mother genotype analysis.

*DHFR* genotype distribution in the group of the 169 mothers (WW = 36.7%, WD = 47.9%, DD = 15.4%) did not differ significantly (*P* = 0.682) from that of their offspring (WW = 40.8%, WD = 46.1%, DD = 13%) and both were in accordance to Hardy–Weinberg equilibrium.

Unexpectedly, the rate of *DHFR*-DD genotype was five-folds higher (DD = 23.8%) in mothers of children with very-early onset than in the same children (DD = 4.8%) (*P* = 0.012), while in the remaining cohort, the mother/child genotype ratio was balanced (12.6% vs. 15.8%; *P* = 0.471) (Figure 3b). As a consequence, those ALL children with homozygous DD-mother had an increased risk to have early-onset (adjusted OR = 3.08; 1.1–8.6; *P* = 0.03). 

No significant differences were instead observed in *MTHFR* 1298 mother/child genotype ratio, though *MTHFR* 1298-CC genotype was three-fold higher (CC = 7.1%) in mothers of children with very-early onset than in the same children (CC = 2.3%), while in the remaining cohort, the mother/child genotype ratio was balanced (8.6% vs. 9.5%) (Figure 3b and Table S3).

### 3.4. Survival Analyses in ALL Children According to DHFR/MTHFR Genotypes

To evaluate possible effects of *DHFR* or *MTHFR* gene also in the childhood life of the ALL cohort, starting from the date of leukemia diagnosis, we performed a survival analysis after a mean follow-up period of about five years.

**Figure 4:**
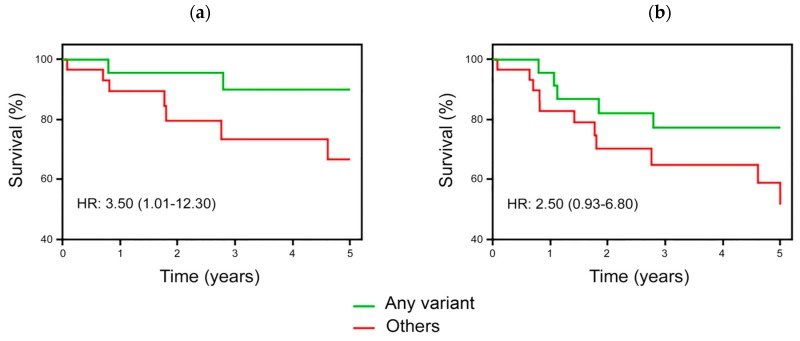
Event-free survival (EFS) among high-risk patients stratified by *DHFR*/*MTHFR* 677 gene variant. EFS at 5-years survey in patients carrying *DHFR* DD- or WD-genotype, and/or *MTHFR* TT-genotype (Any variant) vs. the remaining patients (Others). Panel (**a**) indicates EFS, as specified in Methods section; Panel (**b**) indicates EFS including prednisone response. The inclusion of the *MTHFR* A1298C variant, or its coupling with *DHFR* ins/del variant, did not modify survival analysis.

The whole cohort of patients underwent treatment defined by the AIEOP protocol [12,29], the mean time of follow-up was 4.6 ± 1.2 years, and overall 10.7 % and 17.5% of children experienced an adverse event in terms of EFS endpoints, as detailed in the methods section. No significant association between a particular genotype and any endpoint was found when all patients were considered. On the contrary, after final risk-based stratification, *DHFR* and *MTHFR* polymorphisms were associated with definite outcome in the high-risk subgroup. In detail, the presence of at least one polymorphic allele in *DHFR* gene (i.e., *DHFR* DD- or WD-genotype) and/or the presence of the *MTHFR* 677 TT-genotype was associated with better prognosis at 5-years follow-up exclusively among high-risk patients (Figure 4). Accordingly, by comparing those cases carrying at least one polymorphic allele, as defined above, (i.e., Any variant) vs. the remaining subgroup of patients (i.e., Others), the latter group had a higher chance to experience an adverse events during the five-year follow-up (HR: 3.5; 1.01–12.3; *P* = 0.05). When prednisone response was included in the survival analysis, the risk associated with EFS was quite similar (HR: 2.5; 0.93–6.8; *P* = 0.06). The paucity of events recorded in the follow-up led to borderline statistical significance, affecting any attempt to identify single gene-specific association or uncover additive or synergistic effects of the two genes. Nonetheless, in an explorative attempt, we separately analyzed the two genes and the respective survival analyses have been reported (Figure S1). Though a statistical significance was never reached, direct relations between the presence of polymorphic alleles and survival were observed for all three gene variants in term of EFS showing interesting stepwise trends as the number of the polymorphic variant allele increased in the genotype of children. In summary, the absence of variant alleles increased the risk of adverse events up to almost four-folds during the follow-up (Figure S1).

## 4. Discussion

Several studies and meta-analyses on the role of folate pathway gene variants in pediatric and adult acute leukemia focused on risk disease, treatment efficacy, and survival disclosing interesting pharmacogenetics considerations [36,37,38]. Less investigated in pediatric cancers is instead the mother-child dyad or parent-child triad approach [39,40,41,42]. The present study was conducted within the frame of the *GEMCDS* study aimed to recognize any genetic and epigenetic factor with prognostic role and effect on any aspect of childhood ALL. In the present study, the genetic side related to the main folate pathway gene variants was dealt with, investigating in detail the role of combined *DHFR* and *MTHFR* gene variants in children with ALL and their mothers to find out potential dyad relationships.

The core result in our survey was that selected gene variants influenced ALL onset age with a gene dosage effect. Interestingly, the same gene variant tunes ALL onset in the opposite direction when stratified by children’s or mother’s genotype, suggesting interesting genetic/epigenetic mother-child mutual cross talks worthy of being further investigated in future researches.

In detail, when the whole cohort of children was stratified by *DHFR* or *MTHFR* 1298, the opposite homozygotes had a significant gap of more than two-years in ALL onset, ascribing to those polymorphic homozygotes a delayed mean onset. On the contrary, *MTHFR* 677 TT-homozygotes experienced ALL one-year earlier, and this could be in part explained by the strong linkage disequilibrium between *MTHFR* 677 T-allele and 1298 C-allele.

On the contrary, the stratification by mother’s genotype *MTHFR* 677 seemed to influence ALL onset; thus, the offspring of mothers carrying 677 T-allele experienced ALL more than one-year and half earlier. The contrasting effect *DHFR* and *MTHFR* genes had on ALL onset prompted us to investigate in detail different gene variant combinations in children, mothers, and in dyad pairs.

Firstly, by mutual genotype stratification, we coupled *DHFR* and *MTHFR* in children to find out any combination responsible for possible additive effects on ALL onset age. Interestingly, the delayed onset reserved to the *DHFR* D-allele was inversely related to the presence of the *MTHFR* 677 T-allele, while due to *disequilibrium*, *MTHFR* 1298 C-allele did not counteract the effect. Accordingly, the greatest act of DD-genotype was observed among *MTHFR* homozygotes (677CC or 1298AA), conversely to what observed in children as the number of *MTHFR* polymorphic-alleles increased in their genotype.

On the contrary, in mothers, the combination of *DHFR* and *MTHFR* genotype tuned disease onset in the opposite direction, reserving the earliest onset to offspring of *DHFR* DD-homozygous mothers in combination with *MTHFR* 677 CC-genotype. Although in opposite directions, children’s and mother’s *DHFR*-genotype had a strong impact on ALL onset, and this was more evident among *MTHFR* 677 CC-homozygotes. Given that a large part of children had their mother enrolled in the study, we stratified ALL onset age of the 169 dyads and performed *DHFR* and *MTHFR* genotype analyses.

Mother’s *DHFR*-genotype and *MTHFR* 1298 genotype by themselves did not seem to influence ALL onset, on the contrary to what observed in children, while mother’s *MTHFR*-677 genotype influenced ALL onset more than children’s *MTHFR*-genotype.

Further combined analyses in dyads disclosed interesting mother-child genotype interactions, highlighting that the strongest genotype combination, in terms of delayed onset, was among children *DHFR* DD-homozygotes of mothers *MTHFR* 677 CC-homozygotes accounting for a mean gap of more than 5.5 years. Of note, this mark was also confirmed comparing any children *DHFR* D-carrier having mother *MTHFR* 677 CC-homozygotes with the rest of child-mother coupling, accounting for a significant mean gap of almost 3.5 years. This evidence supports the concept that *DHFR* and *MTHFR* variants have opposite effects on disease onset depending on the mother rather than the child being the carrier. Accordingly, the combination of DD-homozygous offspring of DD-homozygous mothers did not have the expected additive effect on delayed onset age.

Moreover, interesting findings were obtained by weighing against the relative frequency of *DHFR* genotypes in mothers and children stratified by onset age quartiles. This analysis revealed an imbalance in *DHFR* genotype distribution, providing a significant underrepresentation of DD-homozygotes among children within the very-early onset group when compared with the remaining quartiles. Conversely, mothers of children with very-early onset carried the highest rate of DD-homozygosis, suggesting that among the very-early onset group, *DHFR* variant inversely clustered in mothers and children.

To better define the observed discrepant role of *DHFR* D-allele on ALL onset age, we should have genotyped father-mother-child triads, also to fully disclose a possible parent-origin-effect (POE). Starting from the evidence that the mean age of ALL onset significantly increased as the number of D-allele increased in the genotype of children, and that this was lost as the number of D-allele increased in the genotype of the mother regardless the child genotype, we suspected POE. In a recent family-based triad study on fetus anencephaly, POE was suspected for *DHFR* ins/del variant. Authors found an increased maternal risk of anencephaly fetus in mother DD-homozygotes, as well as in children DD-homozygotes, and POE analysis demonstrated a higher risk of maternal transmission of the D-allele compared with paternal transmission [23].

By lacking the paternal genotype in our analysis, we can only suspect POE by observing that WD-carrier children with certain paternal D-allele origin (i.e., those with WW mother) had a delayed onset when compared with WD children of DD mothers. Besides, among WD-heterozygous offspring, none of those with WW-homozygous mother had ALL before 6-year (7.3 years mean age), while those with DD-homozygous mother experienced ALL before 5-months (5.5 years mean age). Similarly, POE could in part be suspected considering the offspring of WD-heterozygous mothers. Those children with at least one D-allele of paternal origin (i.e., DD-homozygous offspring) had delayed onset differently to those WD-heterozygous children of DD-mothers. Finally, we cannot completely discriminate the paternal/maternal origin of the D-allele among the WD-offspring, neither the paternal D-allele condition (i.e., heterozygotes or homozygotes) but only exclude maternal D-allele origin or confirm maternal contribution among DD-children.

Alternatively, we can hypothesize that the presence of unfavorable in utero environment in mothers carrying DD-genotype (e.g., anomalous folate isoform pool) might predispose to early child cancer development.

Unbalanced folate homeostasis during pregnancy may cause adverse epigenetic mechanisms on the offspring [43], and though maternal folate supplementation reduces congenital malformations [24], indiscriminate fortification may cause a different kind of pediatric cancers [21,24,44,45,46,47,48].

Childhood ALL is not considered congenital cancer, although sentinel somatic genomic lesions are assessable at birth occurring in utero during fetal hematopoiesis [6]. Primarily for cases with very-early onset, ALL origins in utero and peculiar inherited predispositions, mutual cross-talk, and epigenetic pathways contribute to leukemogenesis [49,50]. Accordingly, secondary genetic events occur postnatally, influencing the variable and protracted latency of cancer [6]. Altogether, this partly accounts for the higher rate of any translocations we found among ALL children within the early-onset age quartiles.

Moreover, the observation in our cohort among the very-early ALL onset group, *DHFR* genotype differently distributed in the child-mother dyad, might suggest that offspring could have epigenetically suffered the in utero mother environment during fetus development.

Some studies on folic acid supplementation focused on either preventing or promoting adult or pediatric cancer [44,48,51], and human tissue cultures grown in folate depletion can result both in expected global hypomethylation and targeted hypermethylation of selected locus [52,53,54]. It is unclear whether folic acid supplement may predispose to carcinogenesis, rather folate unbalancing may affect genome integrity and make cells prone to neoplastic transformation [55,56,57]. Epigenetic modifications under folate unbalance are irreversible and cannot be recovered. The folate dual effect, known as “double-edged sword”, arises by the cooperation of specific gene polymorphisms, folate pool availability, and homocysteine levels [19,58]. During the embryo development, maternal folate pool necessarily plays key epigenetic roles. In this context, the same group of authors found an increased childhood retinoblastoma risk associated with folic acid fortification in mother with polymorphic *DHFR* gene [21], and that food-derived folate during pregnancy protected against childhood retinoblastoma [59]. Besides, a higher intake of folic acid is associated with circulating unmetabolized folic acid (UMFA), which has raised some controversies in health concerns [46,60]. Diminished capacity in converting folic acid into fully active folate, as in the case of *DHFR* and *MTHFR* gene variants, affects UMFA threshold particularly for pregnant women [61]. It is to note that virtually all mothers recruited in our survey took folate supplement at the time of pregnancy, and this aspect deserves dedicated pharmacogenetics investigations.

Although plausible, any epigenetics mechanism hypothesized in the present research is not supported by experimental data, and epigenetics is far from the primary objective of the present paper. Although in pediatric cancers, the reasonable focus has directed toward the affected child, the impact of maternal genetics via in utero environment should be considered as potentially affecting the fetus hematopoiesis and leukemogenesis. Intracellular and circulating folate balancing depends on one-carbon metabolism that is controlled by polymorphic genes that tune one-carbon derivatives optimizing DNA synthesis, methylation, and cell proliferation, targeting cell-specific gene expression during embryogenesis. Besides, chromatin methylation, chromosome stability, and fidelity in DNA neo-synthesis strongly depend on folate derivatives and specific gene variants [19,62].

Accordingly, maternal gene variants may unbalance the in utero folate isoform availability and alter fetal hematopoiesis. In several investigations, it has been demonstrated that among case mothers but not among fathers or affected children, folate gene variants were associated with child anomalies as pediatric anencephaly, Down syndrome, autism, or brain defects, and this strongly fits with the in utero origin hypothesis of childhood ALL [6,63,64].

Moreover, hypomethylation of maternal DNA from affected mothers was found when compared to control mother DNA, and circulating homocysteine, adenosine, and S-adenosylhomocysteine were unbalanced in case mothers consistent with reduced methylation capacity. Altogether, this suggests that maternal genetics/epigenetics may influence fetal cancer predisposition with a possible effect on cancer latency during childhood life [65].

Finally, the association between *DHFR* or *MTHFR* gene variants and survival in hematological malignancies has been widely reported both for an adult [15,17,18,19,66] and pediatric leukemia [11,25,26,27]. Moreover, particular haplotypes in the promoter of *DHFR* have been previously found useful in predicting outcome in ALL children under Dana-Farber Cancer Institute (DFCI) ALL Consortium protocol ([27,67] and more recently in AIEOP-protocol [26]. In this context, the existence of strong linkage disequilibrium between two *DHFR* promoter polymorphisms (i.e., −317AG, rs408626, and the ins/del, rs70991108) [67] may account for the good prognosis we observed among *DHFR* DD-homozygous ALL children during the five-year follow-up. For that reason, considering that in the majority of cases, the −317G-allele contains one 19bp D-allele, the absence of adverse events we found among DD-homozygotes during the follow-up in AIEOP protocol completely matches with the absence of adverse events Krajinovic and colleagues observed in a ten-year follow-up for those −317GG-homozygotes under the AIEOP protocol [26]. Finally, also additional *MTHFR* haplotypes, mainly considering the existing disequilibrium between 677 T-allele and 1298 C-allele, are plausible candidate targets together with those in the *MTHFR* promoter and are worthy of dedicated investigations. Accordingly, extended maternal haplotypes for both *DHFR* and *MTHFR* genes should be included in future analyses.

## 5. Conclusions

In summary, folate pathway gene variants play pleiotropic roles in the context of pediatric cancers. Together with the well-known pharmacogenetics effects on cancer risk and survival, we here reported a novel potential role of *MTHFR* and *DHFR* gene variants in determining and predicting the time of leukemia onset, adding new insights on the complex genetic and epigenetic mechanisms responsible for the wide postnatal latency of childhood leukemia. Considering the multiple interactions existing among genetically inherited predispositions, somatically acquired alterations, and the environment in leukemogenesis and cancerogenesis, pediatric cancers get together these features in the mother/embryo in utero milieu. Accordingly, case-parent trios or mother-child dyads genetic background and haplotypes should be always considered for a comprehensive understanding of the genetic and molecular bases of childhood cancers or when evaluating putative susceptibility loci. Finally, to validate the results from the present study, they should be confirmed in a larger parent-child population, also considering additional gene variants.

## Figures and Tables

**Table 1:** Demographic and clinical features of patients stratified by gender.

Demographic and Clinical Features	Whole Cohort *n* (%)	Male *n* (%)	Female *n* (%)	*P*
*n* (%)	235 (100)	127 (54)	108 (46)	ns
**Age at onset (years)**				
Mean ± SD	6.13 ± 4.30	6.53 ± 4.45	5.66 ± 4.03	0.06
Range	0.003–16.9	0.003–16.9	0.068–16.81	
Peak	2.9	3.0–3.5	2.5–3.5	
<10 years	184 (78.3)	95 (51.6)	89 (48.4)	ns
≥10 years	51 (21.7)	32 (62.7)	19 (37.3)	
**ALL phenotype**				
B-phenotype	204 (86.8)	106 (52)	98 (48)	0.03
T-phenotype	27 (11.5)	20 (74)	7 (26)	
Other*	4 (1.7)	1 (25)	3 (75)	-
**DNA index** (*n* = 192)				
≥1.16	41 (21.3)	22 (53.6)	19 (46.4)	ns
<1.16	151 (78.6)	84 (55.6)	67 (44.4)	
not available	43	21	22	-
**Translocations**				
Bcr-Abl1/t(9;22) (y/n; *n* = 209)	3/206 (1.43)	3/112 (2.6)	0/94 (0)	ns
t(4;11) (y/n; *n* = 207)	3/204 (1.45)	0/115 (0)	3/89 (3.26)	0.08
**Any translocation:** t(9;22); t(4;11); t(12;21); t(1;19); t(9:11) (y/n; *n* = 209)	49/160 (23.4)	28/86 (24.6)	21/74 (22.1)	ns
**Response to prednisone**				
Prednisone good responders	217 (92.3)	114 (52.5)	103 (47.5)	0.06
Prednisone poor responders	16 (6.8)	12 (75)	4 (25)	
Unknown	2 (0.85)	1 (50)	1 (50)	-
**Final risk stratification** (*n* = 235)				
High-risk (HR)	53 (22.5)	31 (58.6)	22 (41.4)	ns
Intermediate-risk (IR)	125 (53.2)	71 (56.8)	54 (43.2)	
Standard risk (SR)	57 (24.3)	32 (56.1)	25 (43.9)	
**Relapses** (*n* = 228)				
Yes	20 (8.8)	12 (60)	8 (40)	ns
No	208 (91.2)	111 (53.4)	97 (46.6)	
**Death in continuous complete remission**	9 (3.8)	6 (66.7)	3 (33.3)	-
After chemotherapy	3 (1.3)	3 (100)	0 (0)	ns
After stem cell transplantation	6 (2.5)	3 (50)	3 (50)	
**Continuous complete remission**	226 (96.2)	121 (53.5)	105 (46.5)	

* Other indicates *n* = 2 Mixed-phenotype acute leukemia (MPAL); *n* = 1 Biphenotypic acute leukemia (BAL); *n* = 1 Acute undifferentiated leukemia (AUL). *P*-comparisons performed between males and females.

**Table 2:** Age of ALL (acute lymphoblastic leukemia) onset according to different children and mothers genotype.

Genotype Groups		Onset Age in Children with That Genotype (Whole-Cohort) Mean ± SD; *Median* (*n*) [Range]	*P*	Onset Age in Children with That Genotype (Dyad-group) Mean ± SD; *Median* (*n*) [Range]	*P*	Onset Age in Children with Mother Carrying That Genotype Mean ± SD; *Median* (*n*) [Range]	*P*
***DHFR*** **ins/del**	WW	5.2 ± 3.7; *4.07* (91) [0.003–16.6]	**0.002 ^a^** **0.0034 ^b^** **0.03 ^c^**	4.8 ± 3.6; *3.8* (69) [0.003–16.6]	**0.007 ^a^** **0.0037 ^b^** **0.033 ^c^**	5.8 ± 4.5; *3.92* (62) [0.7–16.6]	ns ^a^ ns ^b^ ns ^c^
	WD	6.7 ± 4.4; *5.04* (108) [0.07–16.8]		6.4 ± 4.5; *4.8* (78) [0.07–16.8]		5.9 ± 4.1; *4.38* (81) [0.003–16.9]	
	DD	7.5 ± 4.8; *5.77* (36) [0.5–16.9]		7.2 ± 4.7; 5.4 (22) [2.05–16.9]		5.8 ± 4.3; *3.72* (26) [0.8–15.1]	
***MTHFR*** **677**	CC	6.4 ± 4.4; *4.66* (85) [0.5–16.7]	ns ^a^ ns ^b^ ns	5.82 ± 4.3; *4.1* (50) [0.67–16.7]	ns ^a^ ns ^b^ ns ^c^	7.0 ± 5.3; *4.27* (46) [0.2–16.9]	**0.057 ^a^** **0.017 ^b^** ns ^c^
	CT	6.2 ± 4.6; *5.23* (104) [0.003–16.9]		6.21 ± 4.8; *4.04* (82) [0.003–16.9]		5.5 ± 3.9; *4.09* (89) [0.068–16.8]	
	TT	5.5 ± 3.2; *4.5* (46) [0.77–15]		5.19 ± 2.8; *4.4* (37) [0.77–11.6]		5.4 ± 3.3; *4.07* (34) [0.003–11.7]	
***MTHFR*** **1298**	AA	5.78 ± 4.15; *4.07* (97) [0.48–16.9]	**0.006 ^a^ 0.005 ^b^** **0.05 ^c^**	5.79 ± 4.21; *4.07* (71) [0.77–16.92]	**0.05 ^a^** **0.04 ^b^** ns ^c^	5.9 ± 4.1; 4.3 (88) [0.003–16.3]	ns ^a^ ns ^b^ ns ^c^
	AC	5.96 ± 4.22; *4.49* (110) [0.003–16.8]		5.76 ± 4.25; *4.21* (85) [0.0027–16.82]		5.85 ± 4.4; *4.1* (67) [0.07–16.8]	
	CC	8.03 ± 4.82; *7.60* (28) [1.85–16.21]		7.05 ± 4.84; *4.09* (13) [1.85–15.13]		6.0 ± 4.7; *3.5* (14) [1.3–15.3]	

*P*^a^ indicates comparisons performed between opposite genotype conditions by T-test (i.e., *DHFR* WW vs. DD; *MTHFR* 677 CC vs. TT, and *MTHFR* 1298 AA vs. CC); *P*^b^ indicates comparisons performed between *DHFR* D-allele carriers vs. WW-homozygotes (dominant model) and *MTHFR* 677 TT- or 1298 CC-homozygotes vs. the remaining genotypes (recessive model) by *T*-test; *P*^c^ indicates *P*-values adjusted for multiple testing.

**Table 3:** Age of ALL onset according to different combinations of children genotypes.

Genotype/Groups (*n* = 235)			*DHFR*				
			WW	WD	DD	*P* ^a^	*P* ^b^
**The onset age of children with that genotype**		Mean ± SD *median* (*n*)	5.2 ± 3.7 *4.07* (91)	6.7 ± 4.4 *5.04* (108)	7.5 ± 4.8 *5.77* (36)	**0.002**	**0.0034**
***MTHFR*** ***677***	***CC***	6.4 ± 4.4 *4.66* (85)	5.5 ± 3.6 *4.21* (35)	6.5 ± 4.5 *5.16* (36)	8.1 ± 5.7 *7.14* (14)	**0.034**	0.069
	***CT***	6.2 ± 4.6 *4.23* (104)	5.1 ± 4.3 *3.78* (39)	6.6 ± 4.6 *5.10* (52)	7.8 ± 4.8 *5.86* (13)	**0.038**	**0.026**
	***TT***	5.5 ± 3.2 *4.5* (46)	4.7 ± 2.1 *4.4* (17)	5.9 ± 3.9 *4.34* (20)	6.2 ± 3.4 *5.68* (9)	0.21	0.09
***MTHFR*** ***1298***	***AA***	5.78 ± 4.15 *4.07 (97)*	4.64 ± 3.46 *3.74* (37)	6.24 ± 4.18 *4.60* (43)	7.07 ± 5 *5.27* (17)	**0.0217**	**0.016**
	***AC***	5.96 ± 4.22 *4.49* (110)	5.35 ± 3.71 *4.25* (44)	5.90 ± 4.4 *4.51* (51)	7.97 ± 4.67 *6.19* (15)	**0.0156**	0.10
	***CC***	8.03 ± 4.82 *7.60* (28)	6.43 ± 4.48 *4.06* (10)	9.28 ± 4.58 *10.25* (14)	7.66 ± 6.43 *6.08* (4)	0.34	0.09

*P^a^* indicates comparisons performed between opposite *DHFR* genotype conditions by *T*-test analysis (i.e., WW vs. DD). *P^b^* indicates comparisons between *DHFR* D-allele carriers vs. WW homozygotes by *T*-test analysis.

## Supplementary Materials

Supplementary materials are available online at https://www.mdpi.com/2073-4425/10/9/634/s1. Table S1: Primer sequences, restriction-product characteristics, and PCR conditions, Table S2: Age of ALL onset according to different combinations of child and mother genotype, Table S3: *DHFR/MTHFR* genotype distribution stratified by ALL onset-age quartiles, Figure S1: Event-free survival (EFS) at 5-years survey in patients according to different *DHFR*/*MTHFR* gene variants considered.
