# Supplementary material for: Maternal Haplotypes in DHFR Promoter and MTHFR Gene in Tuning Childhood Acute Lymphoblastic Leukemia Onset-Latency: Genetic/Epigenetic Mother/Child Dyad Study (GEMCDS)
Source: Genes (Basel). 2019 Aug 22;10(9):634. doi: 10.3390/genes10090634 (PMC6770441; doi:10.3390/genes10090634)
Supplement: Supplementary file 1 [file genes-10-00634-s001.pdf]

genes MDPI

Supplementary Material

Table S1. Primer sequences, restriction-product characteristics and PCR conditions

Gene variants	Oligo sequence	Amplicon size (bp)	Restriction enzyme	Allele detected (bp)	PCR thermal profile
DHFR ins/del (rs70991108)					
Fw<br>Fw<br>Rv	5′-CCACGGTCGGGGTACCTGGG-3′<br>5′-ACGGTCGGGGTGGCCGACTC-3′<br>5′-AAAAGGGGAATCCAGTCGG-3′	113/94	--	(W) 113<br>(D) 94	94°C/5'; 94°C/55", 60°C/55", 72°C/25"; 72°C/5' (35 cycles)
Fw Py<br>Rv Py<br>Sq	5′-CAAGAACGGGGACCTGCC-3′<br>5′-ATCCTCTCGCCGGGAGTC-3′[Bio]<br>5′-GCCCACGGTCGGGGT-3′	133/114	--	--	94°C/5'; 94°C/30", 60°C/30", 72°C/30"; 72°C/5' (35 cycles)
MTHFR C677T (rs1801133)					
Fw Py<br>Rv Py<br>Sq	5′-ATCCCTATTGGCAGGTTACCC-3′<br>5′-AAAGAAAAGCTGCGTGATGATGA-3′[Bio]<br>5′-GAAGGTGTCTGCGGG-3′	121	--	--	94°C/5'; 94°C/30", 54°C/30", 72°C/60′'; 72°C/5' (35 cycles)
Fw Rst<br>Rv Rst	5′-CGAAGCAGGGAGCTTTGAG**T**C-3′<br>5′-AGGACGGTGCGGTGAGAGTG-3′	233	*HinfI* (37°C)	(C) 216+17<br>(T) 176+40+17	94°C/5'; 94°C/45", 65°C/60", 72°C/90"; 72°C/5' (30 cycles)
MTHFR A1298C (rs1801131)					
Fw Py<br>Rv Py<br>Sq	5′- CTGCCCTCTGTCAGGAGTGT -3′<br>5′- CATTCCGGTTTGGTTCTCC -3′[Bio]<br>5′- GGAGCTGACCAGTGAA -3′	222	--	--	94°C/5'; 94°C/30", 57°C/15", 72°C/45′'; 72°C/5' (35 cycles)
Fw Rst<br>Rv Rst	5′-GGGAGGAGCTGACCAGTG**C**AG -3′<br>5′-GGGGTCAGGCCAGGGGCAG-3′	138	*Fnu4HI* (37°C)	(A) 138<br>(C) 19+119	94°C/5'; 94°C/30", 59°C/13", 72°C/17′'; 72°C/5' (30 cycles)

Fw and Rv, indicate the forward and reverse primer respectively; the underlined bold letter in the oligo sequence indicates the mutagenized forward primer; Sq, indicates the specific sequencing primer; Rst and Py, indicate restriction and Pyrosequencing technique respectively; [Bio], indicates the biotinylated primer; W and D, indicate the common and rare (polymorphic) *DHFR* allele respectively; C and T, indicate the common and rare (polymorphic) *MTHFR* 677 allele respectively; A and C, indicate the common and rare (polymorphic) *MTHFR* 1298 allele respectively

Table S2. Age of ALL onset according to different combinations of children and mothers genotype

Genotype Groups (n=169)		*DHFR* ins/del (*children*) WW<br>mean±SD<br>*median* (n)<br>range	WD<br>mean±SD<br>*median* (n)<br>range	DD<br>mean±SD<br>*median* (n)<br>range	P^a<br>P^b
Onset in children with that genotype		4.8±3.6<br>*3.78* (69)<br>0.003-16.6	6.4±4.5<br>*4.82* (78)<br>0.04-16.8	7.2±4.7<br>*5.44* (22)<br>2.1-16.9	**0.007**<br>**0.002**
Mothers (n=169)					
***DHFR* ins/del**	WW (n=62)	5.2±3.8<br>*3.92* (42)<br>1.3-16.6	7.3±5.5<br>*4.18* (20)<br>6.1-16.5	-	**0.04**
	WD (n=81)	4.2±3.1<br>*3.78* (27)<br>0.003-15.9	6.4±4.2<br>*5.49* (41)<br>0.07-16.8	7.8±4.9<br>*5.68* (13)<br>3.2-16.9	**0.005**<br>**0.004**
	DD (n=26)	-	5.5±4.2<br>*3.40* (17)<br>0.4-15.1	6.4±4.6<br>*5.21* (9)<br>2.1-14.3	ns
***MTHFR* 677**	CC (n=46)	6.05±4.6<br>*4.15* (20)<br>0.17-15.9	7.15±5.4<br>*4.66* (21)<br>1.3-16.5	10.1±6.7<br>*10.3* (5)<br>3.1-16.9	0.061<br>0.08
	CT (n=89)	4.17±2.9<br>*3.36* (34)<br>1.3-16.6	6.3±4.4<br>*5.09* (41)<br>0.07-16.8	6.1±3.8<br>*4.79* (14)<br>2.1-16.3	**0.02**<br>**0.007**
	TT (n=34)	4.6±3.0<br>*3.78* (15)<br>0.003-11.6	5.8±3.4<br>*4.3* (16)<br>0.8-11.7	7.6±3.6<br>*7.7* (3)<br>3.7-11.1	0.07<br>ns
***MTHFR* 1298**	AA (n=88)	4.79±3.6<br>*3.7* (38)<br>0.003-16.6	6.0±3.8<br>*5.2* (36)<br>0.77-15.1	8.4±4.8<br>*6.7* (14)<br>3.05-16.9	**0.0029**<br>**0.0166**
	AC (n=67)	4.27±2.8<br>*3.7* (26)<br>0.17-14.65	7.05±5.1<br>*5.1* (35)<br>0.07-16.8	5.8±4.1<br>*4.6* (6)<br>2.1-13.1	ns<br>**0.009**
	CC (n=14)	7.9±5.1<br>*7.5* (5)<br>2.3-15.9	5.5±4.9<br>*2.9* (7)<br>1.23-15.1	2.3±0.2<br>*2.3* (2)<br>2.7-3.1	ns<br>ns

Genotype Groups (n=169)		*MTHFR* 677 (*children*) CC<br>mean±SD<br>*median* (n)<br>range	CT<br>mean±SD<br>*median* (n)<br>range	TT<br>mean±SD<br>*median* (n)<br>range	P^a<br>P^b
Onset in children with that genotype		5.8±4.3<br>*4.10* (50)<br>0.7-16.7	6.2±4.7<br>*4.04* (82)<br>0.003-16.9	5.2±2.8<br>*4.4* (37)<br>0.8-11.6	ns
Mothers (n=169)					
***MTHFR* 677**	CC (n=46)	6.8±4.9<br>*4.47* (26)<br>1.9-16.8	7.2±5.9<br>*3.72* (20)<br>0.2-16.9	-	ns
	CT (n=89)	4.8±3.3<br>*3.24* (24)<br>0.7-12.4	5.9±4.6<br>*4.05* (46)<br>0.07-16.8	5.1±2.6<br>*5.52* (19)<br>1.5-11.6	ns
	TT (n=34)	-	5.6±3.6<br>*4.39* (16)<br>0.003-11.7	5.3±3.1<br>*4.05* (18)<br>0.8-11.1	ns
***DHFR* ins/del**	WW (n=62)	5.9±4.8<br>*3.68* (22)<br>0.7-16.5	6.3±5.1<br>*3.75* (27)<br>1.3-16.6	5.0±2.4<br>*6.06* (13)<br>1.4-9.5	ns
	WD (n=81)	6.0±3.9<br>*4.77* (22)<br>2.3-16.7	6.0±4.7<br>*4.04* (42)<br>0.003-16.9	5.6±2.8<br>*4.4* (17)<br>2.8-11.1	ns
	DD (n=26)	5.0±3.8<br>*2.72* (6)<br>2.14-10.7	6.8±4.7<br>*5.86* (13)<br>2.0-15.1	4.5±3.8<br>*3.05* (7)<br>0.8-11.6	ns
***MTHFR* 1298**	AA (n=88)	6.7±4.2<br>*5.1* (18)<br>2.3-16.7	6.2±4.7<br>*3.9* (43)<br>0.003-16.9	4.75±2.5<br>*4.1* (27)<br>0.77-11.1	**0.03**<br>**0.04**
	AC (n=67)	4.8±4.1<br>*3.2* (24)<br>0.66-16.5	6.46±4.8<br>*4.51* (33)<br>0.07-16.8	6.4±3.2<br>*6.4* (10)<br>1.3-11.6	ns<br>ns
	CC (n=14)	6.74±4.3<br>*6.1* (8)<br>2.7-15.1	4.98±5.4<br>*2.99* (6)<br>1.29-15.9	-	-

Genotype Groups (n=169)		*MTHFR* 1298 (*children*) AA<br>mean±SD<br>*median* (n)<br>range	AC<br>mean±SD<br>*median* (n)<br>range	CC<br>mean±SD<br>*median* (n)<br>range	P^a<br>P^b
Onset in children with that genotype		5.79±4.21<br>*4.07* (71)<br>0.77-16.92	5.76±4.25<br>*4.21* (85)<br>0.003-16.82	7.05±4.64<br>*4.09* (13)<br>1.85-15.13	ns<br>ns
Mothers (n=169)					
***DHFR* ins/del**	WW (n=62)	5.38±4.17<br>*3.76* (28)<br>1.32-16.64	6.07±4.66<br>*4.10* (28)<br>0.66-16.5	7.25±5.57<br>*5.41* (6)<br>1.85-15.13	ns<br>ns
	WD (n=81)	6.28±4.22<br>*4.24* (32)<br>2.37-16.92	5.53±4.04<br>*4.51* (43)<br>0.003-16.82	6.54±4.43<br>*4.05* (6)<br>3.15-12.43	ns<br>ns
	DD (n=26)	5.4±4.5<br>*3.4* (11)<br>0.77-15.1	5.84±4.27<br>*3.56* (14)<br>2.03-14.28	8.94 (1)	-
***MTHFR* 677**	CC (n=46)	8.72±5.64<br>*7.86* (14)<br>1.82-16.91	5.97±4.96<br>*3.77* (26)<br>0.17-16.5	7.37±5.46<br>*5.84* (6)<br>1.85-15.13	ns<br>ns
	CT (n=89)	4.91±3.76<br>*3.54* (34)<br>1.32-16.65	5.64±4.01<br>*4.38* (48)<br>0.068-16.82	6.76±4.25<br>*4.01* (7)<br>3.16-12.44	ns<br>ns
	TT (n=34)	5.99±3.08<br>*4.07* (23)<br>0.77-11.64	5.78±3.81<br>*4.70* (11)<br>0.003-11.67	-	-
***MTHFR* 1298**	AA (n=88)	5.9±4.1<br>*4.5* (53)<br>0.77-16.9	5.8±4.0<br>*4.2* (35)<br>0.003-14.7	-	-
	AC (n=67)	5.5±4.5<br>*3.3* (18)<br>1.8-15.1	5.9±4.4<br>*4.4* (40)<br>0.07-16.8	6.33±4.58<br>*4.01* (9)<br>1.85-12.65	ns<br>ns
	CC (n=14)	-	4.9±4.4<br>*2.99* (10)<br>1.29-15.93	6.65±5.03<br>*8.26* (4)<br>2.93-15.13	ns<br>ns

P^a, indicates comparisons performed between opposite genotype conditions (i.e. *DHFR* WW *versus* DD; *MTHFR* 677 CC *versus* TT and *MTHFR* 1298 AA *versus* CC); P^b, indicates comparisons performed between *DHFR* D-allele carriers *versus* WW-homozygotes (dominant model) and *MTHFR 677* TT- or *MTHFR 1298* CC-homozygotes *versus* the remaining genotypes (recessive model).

Table S3. *DHFR*/*MTHFR* genotype distribution stratified by ALL onset-age quartiles

Age of onset quartiles			1st		2nd		3rd		4th		2nd + 3rd + 4th	
	Genotype		%	(n)	%	(n)	%	(n)	%	(n)	%	(n)
Children whole cohort (n=235)	*DHFR* ins/del	WW	49.2	(29)	49.6	(23)	40.7	(24)	25.4	(15)	35.2	(62)
		WD	42.4	(25)	33.1	(25)	47.4	(28)	50.9	(30)	47.2	(83)
		DD	8.4	(5)	17.3	(10)	11.9	(7)	23.7	(14)	17.6	(31)
	MTHFR 677	CC	39.0	(23)	31.0	(18)	30.5	(18)	44.1	(26)	35.2	(62)
		CT	47.4	(28)	43.1	(25)	42.4	(25)	44.1	(26)	43.2	(76)
		TT	13.6	(8)	25.9	(15)	27.1	(16)	11.8	(7)	21.6	(38)
	MTHFR 1298	AA	44.1	(26)	43.1	(25)	42.4	(25)	35.6	(21)	40.3	(71)
		AC	50.8	(30)	41.4	(24)	52.5	(31)	42.4	(25)	45.5	(80)
		CC	5.1	(3)	15.5	(9)	5.1	(3)	22.0	(13)	14.2	(25)
Children dyads (n=169)	*DHFR* ins/del	WW	50.0	(21)	45.2	(19)	40.5	(17)	27.9	(12)	37.8	(48)
		WD	45.2	(19)	38.1	(16)	45.2	(19)	55.8	(24)	46.5	(59)
		DD	4.8	(2)	16.7	(7)	14.3	(6)	16.3	(7)	15.7	(20)
	MTHFR 677	CC	33.3	(14	23.8	(10)	31.0	(13)	30.2	(13)	28.4	(36)
		CT	50.0	(21)	52.4	(22)	35.7	(15)	55.8	(24)	48.0	(61)
		TT	16.7	(7)	23.8	(10)	33.3	(14)	14.0	(6)	23.6	(30)
	MTHFR 1298	AA	42.9	(18)	45.2	(19)	40.5	(17)	39.5	(17)	41.7	(53)
		AC	54.8	(23)	42.9	(18)	54.8	(23)	48.8	(21)	48.8	(62)
		CC	2.3	(1)	11.9	(5)	4.7	(2)	11.7	(5)	9.5	(12)
Mothers (n=169)	*DHFR* ins/del	WW	40.5	(17)	35.7	(15)	31.0	(13)	39.5	(17)	35.4	(45)
		WD	35.7	(15)	54.8	(23)	54.8	(23)	46.5	(20)	52.0	(66)
		DD	23.8	(10)	9.5	(4)	14.2	(6)	14.0	(6)	12.6	(16)
	MTHFR 677	CC	23.8	(10)	33.3	(14)	21.4	(9)	37.2	(16)	30.7	(39)
		CT	61.9	(26)	45.3	(19)	61.9	(26)	41.9	(18)	49.6	(63)
		TT	14.3	(6)	21.4	(9)	16.7	(7)	20.9	(9)	19.7	(25)
	MTHFR 1298	AA	50.0	(21)	50.0	(21)	57.1	(24)	51.2	(22)	52.8	(67)
		AC	42.9	(18)	38.1	(16)	38.1	(16)	39.5	(17)	38.6	(49)
		CC	7.1	(3)	11.9	(5)	4.8	(2)	9.3	(4)	8.6	(11)

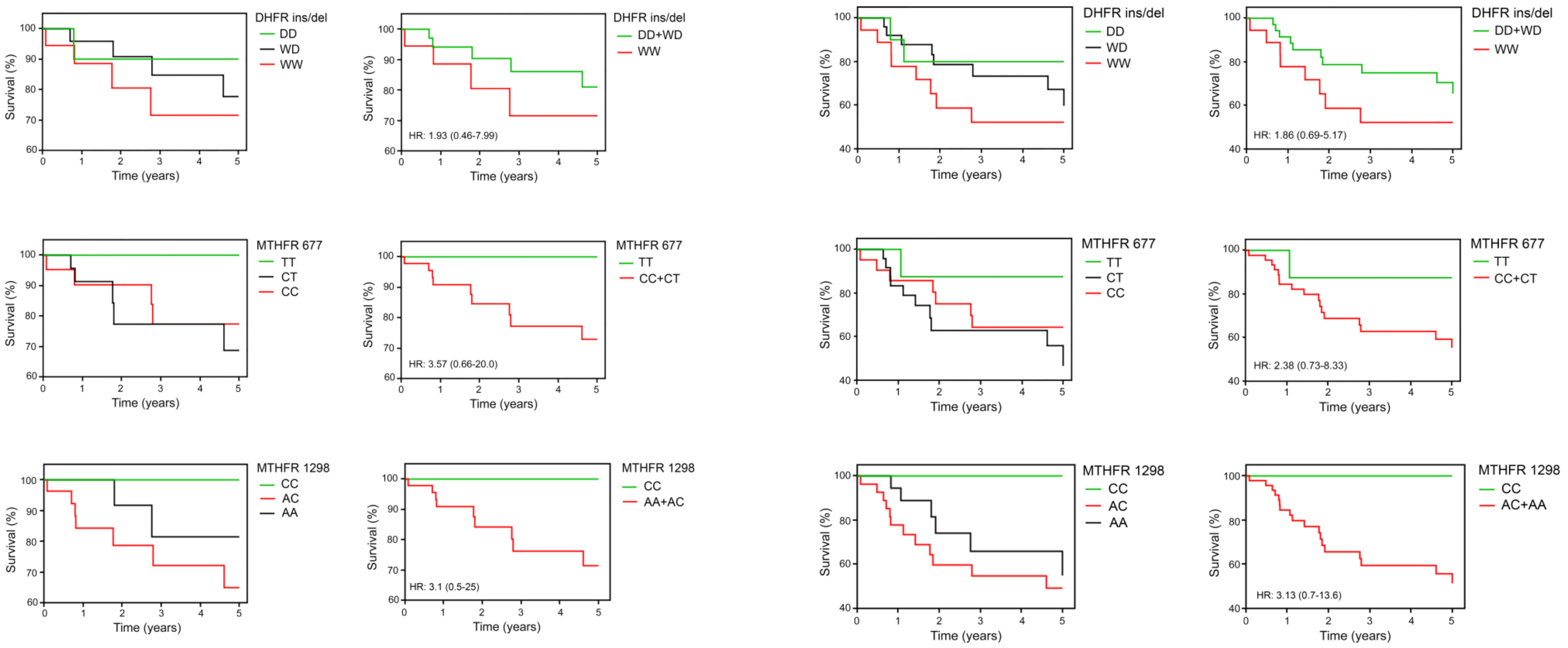

(**a**) (**b**)

Figure S1. Event-free survival (EFS) among high-risk patients at 5-years survey in patients according to different *DHFR*/*MTHFR* variants. EFS curves are provided for each genotype of indicated gene variants. In **A**, EFS according to *DHFR* (**upper panels**) and *MTHFR* (**lower panels**). In **B**, EFS including prednisone response according to *DHFR* (**upper panels**) and *MTHFR* (**lower panels**). Associated HR with 95% CI are indicated. EFS: Event-free survival; HR: Hazard risk.
